# PATIENT VOICES, a project for the integration of the systematic assessment of patient reported outcomes and experiences within a comprehensive cancer center: a protocol for a mixed method feasibility study

**DOI:** 10.1186/s12955-020-01501-1

**Published:** 2020-07-28

**Authors:** Cinzia Brunelli, Claudia Borreani, Augusto Caraceni, Anna Roli, Marco Bellazzi, Linda Lombi, Emanuela Zito, Chiara Pellegrini, Pierangelo Spada, Stein Kaasa, Anna Maria Foschi, Giovanni Apolone, on behalf of the PATIENT VOICES study group, Giovanni Apolone, Marco Bellazzi, Filiberto Belli, Claudia Borreani, Cinzia Brunelli, Giuseppe Capri, Augusto Caraceni, Paolo Casali, Paolo Corradini, Filippo de Braud, Anna Maria Foschi, Secondo Folli, Marina Garassino, Lisa Licitra, Nicola Nicolai, Chiara Pellegrini, Marco Platania, Giuseppe Procopio, Anna Roli, Roberto Salvioni, Pierangelo Spada, Riccardo Valdagni, Emanuela Zito

**Affiliations:** 1 Palliative Care, Pain Therapy and Rehabilitation Unit, Fondazione IRCCS Istituto Nazionale Dei Tumori, Milan, Italy; 2 Clinical psychology Unit, Fondazione IRCCS Istituto Nazionale dei Tumori, Milan, Italy; 3 Quality, education and data protection Unit, Fondazione IRCCS Istituto Nazionale dei Tumori, Milan, Italy; 4 Information and communication technology Unit, Fondazione IRCCS Istituto Nazionale dei Tumori, Via Venezian 1, 20133 Milan, Italy; 5 Department of Sociology, Università Cattolica del Sacro Cuore, Milan, Italy; 6 Nursing, technical and rehabilitation services Unit, Fondazione IRCCS Istituto Nazionale dei Tumori, Milan, Italy; 7 European Palliative Care Research Centre (PRC), Department of Oncology, Oslo University Hospital and Institute of Clinical Medicine, University of Oslo, Oslo, Norway; 8 Patient representative, Scientific Directorate, Fondazione IRCCS Istituto Nazionale dei Tumori, Milan, Italy; 9 Scientific Directorate, Fondazione IRCCS Istituto Nazionale dei Tumori, Milan, Italy

**Keywords:** Patient participation, Patient reported outcome measures, Medical informatics, Implementation science, Oncology

## Abstract

**Background:**

Listening to “patient voices” in terms of symptoms, emotional status and experiences with care, is crucial for patient empowerment in clinical practice. Despite convincing evidence that routine patient reported outcomes and experience measurements (PRMs) with rapid feed-back to oncologists can improve symptom control, patient well-being and cost effectiveness, PRMs are not commonly used in cancer care, due to barriers at various level. Part of these barriers may be overcome through electronic PRMs collection (ePRMs) integrated with the electronic medical record (EMR). The PATIENT VOICES initiative is aimed at achieving a stepwise integration of ePRMs assessment into routine cancer care. The feasibility project presented here is aimed at assessing the knowledge, use and attitudes toward PRMs in a comprehensive cancer centre; developing and assessing feasibility of a flexible system for ePRM assessment; identifying barriers to and developing strategies for implementation and integration of ePRMs clinical practice.

**Methods:**

The project has been organized into four phases: a) pre-development; b) software development and piloting; c) feasibility assessment; d) post-development. A convergent mixed method design, based on concurrent quantitative and qualitative data collection will be applied. A web-survey on health care providers (HCPs), qualitative studies on patients and HCPs (semi-structured interviews and focus groups) as well as longitudinal and cross-sectional quantitative studies will be carried out. The quantitative studies will enroll 600 patients: 200 attending out-patient clinics (physical symptom assessement), 200 attending inpatient wards (psychological distress assessment) and 200 patients followed by multidisciplinary teams (patient experience with care assessment). The Edmonton symptom assessment scale, the Distress Thermometer, and a tool adapted from existing patient reported experience with cancer care questionnaires, will be used in quantitative studies. A multi-disciplinary stakeholder team including researchers, clinicians, health informatics professionals, health system administrators and patients will be involved in the development of potentially effective implementation strategies in the post development phase.

**Discussion:**

The documentation of potential advantages and implementation barriers achieved within this feasibility project, will serve as a starting point for future and more focused interventions aimed at achieving effective ePRMs routine assessment in cancer care.

**Trial registration:**

ClinicalTrials.gov (NCT03968718) May 30th, 2019.

## Background

The recognition of the patients’ perspective as crucial in medical decision making, represents a major shift in medicine during the last decades [1]. Listening to “patient voices” in terms of symptoms, function, emotional status, satisfaction and experiences with treatment and care received, is essential for the empowerment of both patients and their caregivers to become more involved in the care process. This is particularly true for cancer, where people often face difficult decisions due to the complexity of treatment choices, tied to the life-threatening nature of the illness and to its emotional consequences.

The assessment of the patient perception of his/her health condition has been labelled differently, but the terms “Quality of Life” introduced in the 1980s, and the following “Health-related Quality of life”, were for long the most frequently used. In 2006 the U.S. Food and Drug Administration proposed to use the term PROs (Patient Reported Outcomes), later extended into PROMs (Patient Reported Outcomes Measures), to indicate “*any report of the status of a patient’s health condition that comes directly from the patient, without interpretation of the patient’s response by a clinician or anyone else”* [2]. More recently the term PREMs (Patient reported experience measures) has been introduced to indicate patients’ perceptions of their experience of the process of care, rather than outcome. PREMs can evaluate objective experiences (i.e waiting time before appointment), observations of healthcare providers’ behaviour (i.e whether or not a patient was given a due information) or satisfaction for the care received, the latter being more influenced by patient expectations and preferences [3, 4]. For sake of simplicity the terminology “patient reported measures” (PRMs) will be used to jointly indicate PROMs and PREMs in the present paper.

PRMs were initially developed to be applied in clinical research to assess efficacy and effectiveness of medical interventions, but they can also be used in clinical practice to drive medical decisions. While the use of PRMs in clinical research is fairly well established, systematic assessment in clinical practice is not widely implemented in routine care delivery [5–7] and may pose practical challenges, like the burden for healthcare providers in administering questionnaires before or during the medical encounter or the difficulty in interpreting results. Nonetheless, systematic symptom assessment in oncology practice is considered one key element of effective integration between oncology and palliative care [8] and there is now convincing evidence that routine use of PROM with rapid feed-back of results to health care providers (HCP), can improve symptom control, patient well-being, cost effectiveness as well as patient engagement and survival [5, 9–12].

Engagement in health care is related to the involvement of the patients, but also of caregivers, clinicians, and other stakeholders (such as researchers, purchasers, policy makers), recognising great relevance to multidisciplinary collaboration [13]. In particular, patient engagement can be defined as the promotion and support of the active involvement and participation of patients, recognizing them adequate decision-making role within the health care process. Recent studies showed that patients with higher levels of engagement reported better physical and psycho-social outcomes of care [14], and that stakeholder engagement was critical for successful implementation of PROMs in cancer care [15].

Advances in health information technology have promoted the development of electronic tools for the collection of PROMs and PREMs through mobile devices (ePRMs), a number of which are currently used in oncology practice [16, 17]. Such systems may help in overcoming some of the limitations mentioned above, permit distant follow-up of patients who are not hospitalized, promote data sharing among care team members and offer powerful opportunities for their integration with patient data from other sources (i.e physicians reported data from electronic medical record (EMR), laboratory data, administrative and cost data).

It has recently been highlighted that successful integration of ePROMs would require overcoming not only technological and reimbursement barriers but also operational ones like standardized methods for integrating them into the clinical workflow [7]. In order to develop effective integration plans, direct evidence on acceptability and feasibility for patients and HCPs, are needed in disease-specific patient populations and countries. Actually the full potential of EMR will not be achieved without the inclusion and integration of PROMs within the EMR itself [7, 18].

**Fig. 1:**
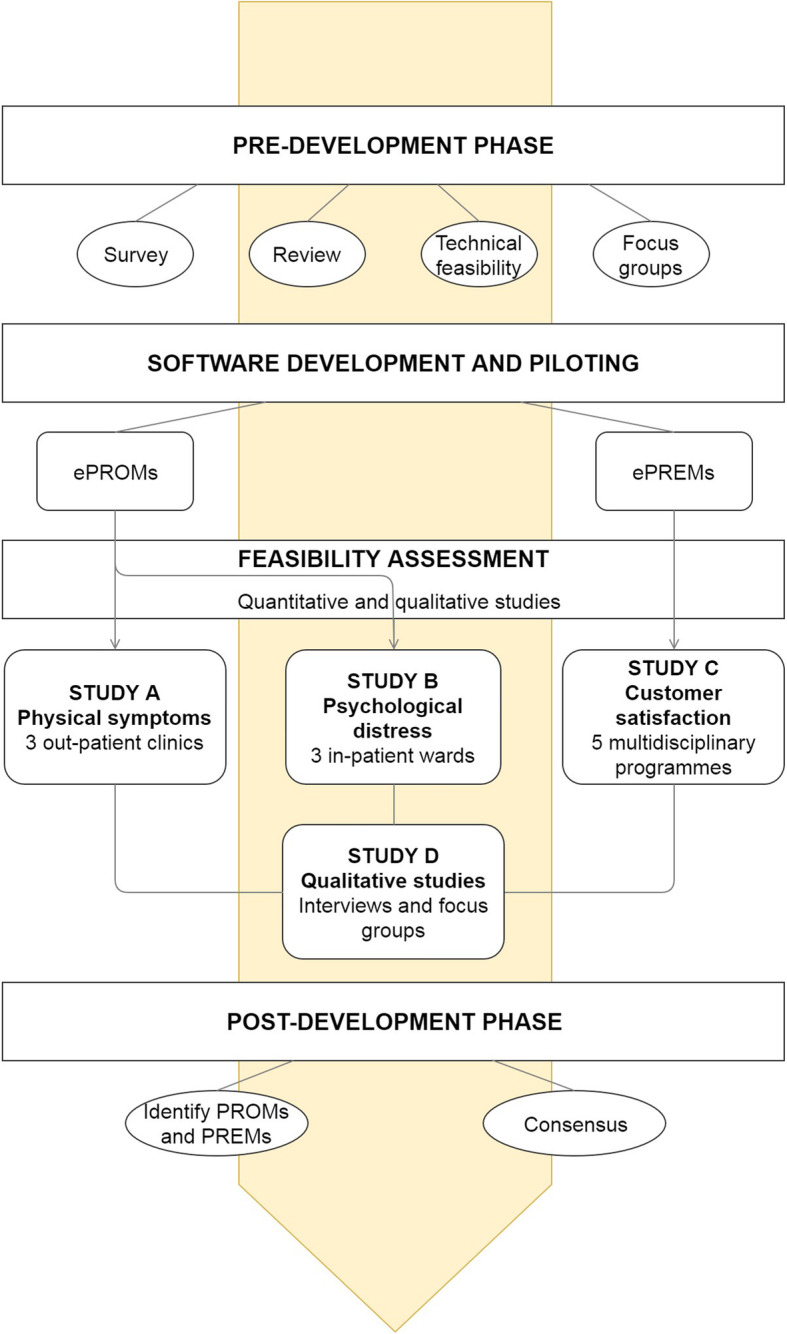
Phases of the PATIENT VOICES feasibility project

At present no standardized PRMs assessment is routinely performed at the Fondazione IRCCS Istituto Nazionale Tumori of Milano (INT) and to the best of our knowledge, no evidence has been published regarding routine PROMs assessment in oncology practice in Italy. The PATIENT VOICES project is aimed at achieving a stepwise integration of PRMs assessment into routine clinical cancer care in our Institute. The feasibility project presented here is the first step in the implementation project. It has been organized into four subsequent phases as reported in Fig. 1:

1. pre-development
2. software development and piloting
3. feasibility assessment
4. post-development phase.

Specific aims and methods of each phase are reported in the following paragraphs.

## Aims and methods

This study protocol has been developed following a convergent mixed method design, based on a concurrent quantitative and qualitative data collection, followed by separated analysis for each method with the aim to obtain different but complementary data on the same topic [19–21] thus gaining a wider understanding of feasibility of PRMs systematic assessment. The quantitative and qualitative strands of the research are performed independently, and their results are brought together in the overall interpretation in order to corroborate and expand our collected data.

### Pre-development phase

The aims of this phase are:

- To assess the attitudes of cancer patients and oncology health care providers towards the routine use of PRMs in cancer care.
  Two focus groups, one with patients and one with clinicians (physicians, nurses and psychologists) will be carried out to explore stakeholders needs, perceptions and attitudes to the use of standardized PRMs assessment in clinical practice.
  A web-survey will be administered to all physicians (medical, radiation and surgical oncologists as well as intensive care specialists), nurses and other health professionals (social workers, physiotherapists and psychologists) employed at our institution (900 to 1000 people); survey remainders will be sent to non-responders until a responder rate of at least 50% will be reached [22]. A sample size of 450 responders will allow to estimate a two-sided 95% confidence interval (CI) for the mean of continuous scores with a precision (half CI width) of 9.2% of its standard deviation [23]. In case of dichotomus response to questionnaire items (yes/no), and in the conservative hypothesis of 50% of “yes”, the precision will be of 4,6% [23]. The research team will develop an ad hoc questionnaire investigating which PRMs are used in clinical practice and in research, and what are the potential advantages and drawbacks of a systematic assessment. Potential advantages and drawbacks of electronic assessment will also be investigated. Data analysis will be mainly descriptive and stratified by professional role.
- To identify and analyze electronic assessment systems developed and used in cancer care.
  A systematic review of the medical literature will be performed in order to find out which systems have been developed for ePRM assessment in cancer care. Eligible systems will be defined as those used in clinical oncology practice settings for any cancer diagnosys (medical, radiation and surgery oncology as well as palliative care and psycho-oncology) which allow the electronic self-assessment of PRMs and summarize patients’ responses to the clinician. Papers reporting evidence from any study design (from feasibility studies to RCT of system implementation) will be considered eligible. The search will be carried out on MEDLINE and EMBASE using both text search and MeSH terms. Google Scholar and Scopus in the previous 5 years will be searched in order to cover a wider set of potentially relevant sources. A search strategy for Pubmed is reported in Table 1. Appropriately revised strategies will be developed for each database. The index of the main health informatics journals covering the previous 3 years will be hand searched. Grey literature (relevant congress abstracts, web sites and press releases) will also be examined. Experts in the field will be contacted in order to identify electronic assessment systems not yet published in the literature. The review will be registered on PROSPERO database [24] and its results will be reported following the PRISMA guidelines for systematic reviews and meta-analyses [25].
  Electronic systems identified will be compared exploring the technical feasibility of ePROMs integration into EMR and identifiyng technological barriers and basic system requirements for their implementation into routine clinical practice. In addition, functional (ie. configuration dashboard, data exportability, data visualization, …) as well as non-functional (ie. integration with other systems, data storage, …) specifications will be examined.

**Table 1:** Search strategy draft to retrieve papers from PubMed

**Query number**	**Query content**
#15	**#1 AND #13 AND #14**
#14	**#6 OR #7 OR #8 OR #9 OR #10 OR #11 OR #12**
#13	**#2 OR #3 OR #4 OR #5**
#12	**Computer [tiab] OR smarphone [tiab] OR web-based OR tablet [tiab]**
#11	**eHealth [tiab] OR mhealth [tiab] OR E-Health [tiab] OR m-health [tiab]**
#10	**“Electronic Health Records”[mh]**
#9	**“Medical Records Systems, Computerized”[mh]**
#8	**“telemedicine”[mh]**
#7	**“Medical Informatics”[mh]**
#6	**“Mobile Applications”[mh]**
#5	**“patient outcome assessment”[mh] OR “patient outcome assessment”[tiab]**
#4	**“quality of life”[mh] OR “quality of life”[tiab]**
#3	**“Patient Reported Experiences”[tiab] OR “Patient Reported Experience”[tiab]**
#2	**“Patient Reported Outcomes”[tiab] OR “Patient Reported Outcome”[tiab]**
#1	cancer [tiab] OR neoplasms [mh] OR tumour [tiab] OR oncol*[tiab] OR carcinoma*[tiab] OR malignan*[tiab]

### Software development and piloting

The objective of this phase is to develop and pilot a prototype system for electronic collection of PROMs and PREMs based upon the results of the the pre-development phase.

**Fig. 2:**
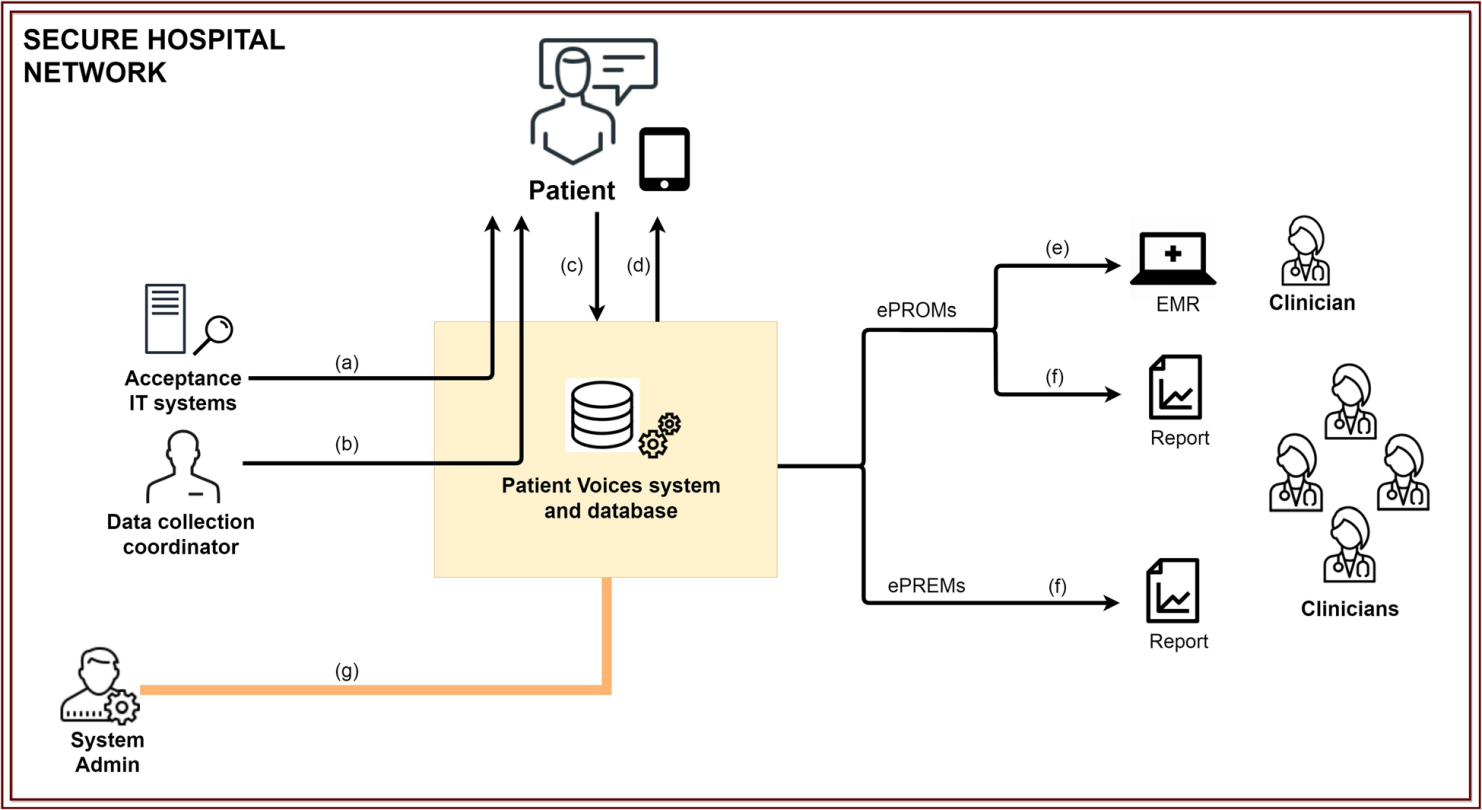
Patient Voices system diagram. **a** and **b** ePROM and ePREM triggers; **c** patient questionnaire completion; **d** feedback to patients; **e** individual level feedback to clinicians; **f** group level feedback clinicians; **g** administrator tasks. (ePROMs: electronic Patient Reported Outcome Measurements, ePREMs: electronic Patient Reported Experience Measurements, EMR: Electronic Medical Record)

The ePRM system is schematically described in Fig. 2. The whole data management process (patient registration, questionnaire completion, data elaboration and clinician consultation) takes place within the hospital network. Expansion of the system allowing also for data completion by the patient from outside the hospital network will be developed in the future. Ideally, PRM triggers will come from hospital acceptance IT systems (i.e. unified booking centre system (UBCS) and admission discharge and transfer system (ADT)) (a) or from a data collection coordinator (b). Then, patients will fill in PRMs questionnaires through a dedicated interface using a tablet provided by the hospital (c); data will be elaborated (scoring and graphical presentation) by the system, and scores will be displayed on the patient interface (d). All data will be saved into a secure server within the hospital network.

A distinction must be drawn between ePROMs and ePREMs as regard how they are reported to clinicians. While individual ePROMs data will be available to the clinicians during the consultation, ePREM data collection should be anonymous to allow the patient freedom in expressiong judgments; for this reason only group level reporting will be available for ePREM and then ePREM system does not need to interact with the EMR. Figure 2 in fact indicates that after completion, individual ePROMs scores are real time transferred and graphically displayed into the ePROMs section of the EMR (e), in order for the clinician to consult them; both ePROMs and ePREMs will be stored and successively elaborated for group level reporting accessible to clinicians (e). Finally Fig. 2 enlights the role of the “system administrator” who operates through a dedicated dashboard to modifiy existing or to add new ePRMs tools, to authorize users to system access, to download patients data and manages group level reports (f).

Based on authors previous experiences and on indications from the literature [17, 26–28], main key features of the system will be:

- ease of accessibility and usage for the patients and clinicians
- security of patient data
- linkage to other systems (EMR, appointment/scheduling, patient portal)
- flexibility (i.e the possibility to create questionnaires without significant programming effort)
- real-time and user-friendly display of ePROMs results to clinicians
- meaningful display of PREMs data for monitoring patient experience with care and for benchmarking between wards

The prototypes will be pilot tested on 5 clinicians and 10 patient advocates. Comments will be collated on: logging in, accessing the system, navigation through the questionnaire; accessing and inspection the results. Staff and patients will be interviewed (semi-structured interviews) and changes will be applied to overcome problems emerged. A new version of the prototype will be developed and used in the quantitative feasibility phase described below (Fig. 1). The system will be further refined for improvement during data collection in feasibility studies. A final version of the system will be developed at the end of data collection and elaboration.

### Feasibility assessment

The aims of this phase are:

- To assess compliance, acceptability, and usability of a routine ePROMs and ePREMs assessment both by patients and health care providers.
- To identify patient and health care provider related barriers.
- To identify potential engagement strategies for the implementation of routine ePROM assessment in oncology clinical practice.

Both quantitative (studies A, B and C) and qualitative (study D) feasibility studies will be carried out:

- STUDY A: a longitudinal study of symptom screening and monitoring in cancer patients attending 3 out-patient clinics of INT, namely palliative care, thoracic oncology and genito-urinary cancer clinics.
- STUDY B: a cross-sectional study of psychological distress screening among in-patients admitted to hematology, medical oncology and breast surgery wards of INT.
- STUDY C: a longitudinal study for the assessment of patient experience of care during different phases of the care process, among patients followed by multidisciplinary teams in INT, namely the Breast Cancer Unit, Prostate cancer program, Thoracic cancer Unit, Head&Neck cancer Unit, Mesenchimal cancer Unit.
- STUDY D: a feasibility qualitative study (interviews with patients and focus groups with HCPs) with users involved in the quantitative studies.

### Methods for quantitative feasibility studies

#### Patient population

All consecutive adult cancer patients attending one of the above out-patient clinics, in-patient wards or followed by one of the multidisciplinary team in a pre-specified time period, will be proposed to be enrolled in the study. Patients with inability to fill in the system due to cognitive impairment, psychological disturbances or language problems as judged by the study personnel upon inclusion, will not be eligible. All physicians working in the outpatient clinic in those days in which system is tested, are eligible to study participation.

#### PROMs and PREMs questionnaires

Grounded on their relevance within patient reported measures literature in oncology [18, 29–32], three PRM areas have been indentified to be addressed in the three quantitative feasibility studies: symptom burden (study A), psychological distress (study B) and patient reported experience and satisfaction with care received (study C).

Symptom burden will be measured using the Edmonton symptom assessment scale (ESAS) [33] (i.e., pain, tiredness, drowsiness, nausea, reduced appetite, breathlessness, depression, anxiety, well-being) plus sleep, constipation and vomiting. The intensity of each symptom is assessed on 0–10 numerical rating scales (higher scores indicate greater symptom severity). The ESAS instrument is one of the most widely used and with a robust psychometric documentation in various languages [33, 34] and has been validated in Italian in a sample of advanced cancer patients [35].

Psychological distress screening among in-patients will be performed using the Distress Thermometer (DT), a single-item 0–10 numerical rating scale, on which participants rate their level of distress from any cause in the preceding 7 days [36]. The DT is accompanied by a 35 item problem list, which prompts patients to identify their problems in five different categories: practical, family related, emotional, spiritual/religious, and physical. Scores of 4 or higher suggest a level of distress that has clinical significance. The DT has been validated in Italian language [37].

In the longitudinal feasibility pilot study C, questionnaires for the survey will be developed from existing instruments (the questionnaire developed in Bench-Can and EURACAN projects [38], the patient satisfaction with cancer care questionnaire [39] and the institutional customer satisfaction survey). The adaptation of the questionnaire content to the specific patient population, will be performed with the contribution of clinicians of the multidisciplinary teams involved, discussed with cancer patients’ representatives and pre-tested among a restricted group of users.

#### Data collection procedures

A trained data collection coordinator (DCC), a research nurse or a psychologist or a physician, will perform eligibility screening, propose the patient to be enrolled in the study, collect written informed consent and provide basic training in how to use the device. The DCC will also be available for any help the patient may need during the compilation. At the end of the compilation, system usability as perceived by patient will be collected and the following data will be registered by the DCC: reason for potential incompleteness, amount of assistance needed and system usability. The system will automatically register clinician access to patients’ data. System usability as perceived by clinicians will be collected at the end of study period.

Patients attending the out-patient clinics involved in Study A, will fill in the symptom burden questionnaire at the first 3 visit performed during the study period, while symptom distress screening among in-patients (Study B) will be performed on admission.

In Study C questionnaires will be filled-in along the care pathway at the following clinically relevant points: T0 (at diagnosis), T1 (first treatment decision making step), T2 (end of active treatment), T3 (disease progression), T4 (follow-up). Exact timing of T0 to T4 will be different for the five multidisciplinary team Units involved and the maximum follow-up time will not exceed 6 months from enrollment.

#### Study endpoints

The main study endpoints are the rates of compliance with the system at baseline assessment by both patient (percent of eligible patients completing electronic questionnaires) and physicians (percent of clinical encounters in which the physician gets access to data provided by ePROM) (the latter only in studies A and B).

Secondary endpoints are:

- proportion of eligible patients among those screened;
- proportion of patients refusing to use the system and reasons for refusal;
- proportion of patients filling in the system without external help;
- average proportion of missing items for each compilation;
- average time to fill in the system;
- average level of perceived system usability by patients;
- average level of perceived system usability by physicians;
- identification of features of the system which need refinement.

Additional secondary endpoints in the longitudinal feasibility studies:

- rate of drop-out during the longitudinal assessment and reasons for drop-out;
- rate of intermittent missing assessment and reasons for missingness;
- clinician perception about the usefulness of PROMs for treatment decisions.

#### Assessment methods

A paper and pencil self-report form will be used to gather patients’ education level and acquaintance/experience with e-devices (smartphones, tablets, computers). Patients’ basic demographic and clinical data (sex, date of birth, tumor site, stage of the disease, pain and other symptom medication prescription) will be gathered from the EMR. Time needed to complete data collection for each patient will be registered by the system. Data regarding gender, years of experience in oncology practice and experience with e-devices, will be collected also for the clinicians participating in the study. After testing, patients and clinicians will fill out the System Usability Scale (SUS), a standardized questionnaire used to assess participants’ perceptions of system usability [29]. The SUS is made up of 10 items rated on a 5-point Likert scale and its final score is a summated rating scale ranging from 0 to 100 after resacaling. An open ended question will also be asked to all participants in order to assess whether any of the system features were difficulty to use or for which they propose modifications.

#### Sample size and data analysis

Six hundred consecutive cancer patients will be enrolled (200 in each of studies A, B, and C).

In the hypothesis that the percentage of successful assessments/consultation (“rate of compliance” among patients and clinicians) is 50% (hypothesis of maximum variability, and then maximum imprecision), a sample size of 200 allows the estimation of a 95% CI for the rate of compliance with a precision (half width) of 6.9% [23].

Basic descriptive statistics will be applied to characterize the study sample. Point and interval estimates (95% CI) of rates and averages described in primary and secondary endpoints, will be calculated for the whole sample and by outpatient clinic/inhospital ward. The association between patient related characteristics and binary as well as continuous outcomes will be respectively analyzed with logistic and linear regression models.

### Methods of qualitative feasibility study

The use of qualitative techniques, namely semi-structured face-to-face interviews and focus groups, is addressed to explore attitudes and opinions about PRMs and ePRMs of both patients and HCPs in the pre-implementation phase, to identify patient and clinician related barriers to adopt ePRM data collection in oncology clinical practice during the feasibility assessment phase (Fig. 1), as well as to pinpoint engagement strategies to promote the use of ePRM system in routine care.

Two separate focus groups (10 patients and 10 clinicians) will be carried out during the pre-development phase of the ePROM system. We opted for two separate focus groups to compare the viewpoints of patients and clinical professionals. After the ePROM feasibility phase, three focus groups, one for each study A, B and C (10 participants each), will also be carried out among clinicians (physicians, psychologists, nurses) who have participated in the three studies. The number of cases in focus groups was determined to support the depth of case-oriented analysis that is fundamental to this mode of inquiry and that would not be possible with wider sample sizes [40], to ensure sufficient heterogeneity between the characteristics of the participants and to allow everyone to intervene in the discussion, avoiding potential frustration due to lack of time to express one’s point of view and opinion [41]. The recruitment strategies will be addressed to ensure heterogeneity with respect to stage of disease (only for patients), professional profiles (only for clinicians) and gender (for both). Thirty semi-structured interviews (enrolling 10 patients for each of study A, B and C) will also be carried out. Purposive sampling will be performed in order to cover a wide range of patient characteristics (sex, age, disease site and stage as well as experience with e-devices and study participation). Feasibility issues addressed during interviews/focus groups will cover aspects common to each study (ease of use of the ePRM, relevance of the content, suggestions for improvement) as well as disease/setting related aspects. A special attention will be dedicated to elderly and to patients with low experience with electronic devices.

Experts in qualitative methods will be engaged both for training and supervision of interviewers that will be enrolled among volunteers and trainees at INT. Data collected will be analysed through T-Lab, a linguistic, statistical and graphical software designed to allow text analysis of qualitative data.

### Post-development phase

The aim of this phase is twofold: to identify specific questionnaires and to develop strategies for the implementation of ePRM systematic assessment in routine oncology care at our Institute (Fig. 1).

A variety of questionnaires are available for the assessment symptom burden, psychological distress, side effects of cancer treatment, experience with care received and quality of life. The choice is to be based on aim of assessment, feasibility and possibility of intervention after assessment. A consensus meeting will be carried out after the feasibility phase is concluded to decide upon which tools to use during the future implementation phase of the PATIENT VOICES project. Representatives of all the clinical and research wards of the INT as well as the research personnel involved in the present project will be invited to participate in the consensus.

Like any change in clinical processes, successful implementation of electronic patient reported assessment in routine clinical workflow, requires institutional commitment from leadership, buy-in from administrators as well as the involvement of multiple stakeholders along the development process. For this reason a multi-disciplinary stakeholder team (MDST) including researchers, oncology clinicians, health informatics professionals, health system administrators and patients will be involved in the development of potentially effective implementation strategies.

The MDST will examine evidence from the literature, results of the survey and of the feasibility studies to answer the following questions:

- How, where, and with what frequency will ePRMs be administered?
- How can users be trained and engaged?
- How will the ePROM-EMR system interaction be governed?
- How will ePROM and ePREM data be acted upon?
- What are the ethical and legal implications of the systematic assessment?

Discussion and consensus among MDST members on the topics above, will be achieved via both meeting participation and written document circulation.

## Data protection issues

Confidentiality issues regarding the registration, handling and storage of data will be a major concern during the system development process. Solution will adhere to current national and European data protection regulations, in particular in accordance with Regulation (EU) 2016/679 of 27 April 2016 (General Data Protection Regulation). Data communication between the device used for data entry and the storage server will be secured using HTTPS over SSL. Appropriate authentication systems will ensure verification of the user identity. Coded PROMs data collected by electronic devices will be transferred to and stored on secure servers hosted by INT. Data will be pseudonimized and encrypted, access logs registered and managed in compliance with GDPR. Patient usernames (codes) that will be used for linking these data to patient’s individual EMR, will also be imported separately and stored into a database on secure servers hosted by INT.

## Discussion

Despite the moral imperative to assess and address to the issues that matter to people with cancer as part of their routine clinical care, PRMs are not commonly used in clinical practice in oncology and awareness of their clinical usefulness is lacking both among HCPs and patients. Admistering and filling in PRMs questionnaire is often considered as “paper work”, feedback about self-assessments are normally not provided to patients, and patients themselves often do not expect to receive any. On these bases it is difficult that PRMs can be usefully implemented as clinical tools, despite recent evidences of their positive impact on patient care [9–12].

A number of initiatives/projects have been proposed to promote systematic assessment of PRMs in clinical practice. The innovation of the PATIENT VOICES project lays in redefining organizational procedures for the administration of PRMs tools in clinical practice, based on the methodological experience from PRMs implementation in research, on the use of IT technology and on a wide engagement of patients, clinicians, researchers and health system administrators for the definition of new procedures. PRM implementation in clinical practice is in fact a complex intervention, which requires change in behaviours in all stakeholders involved and this is the reason why the project puts such emphasis in studying feasibility and developing implementation strategies finalized to overcome potential barriers at various levels [42].

The PATENT VOICES project is extensively based on the use of electronic devices, in line with the present trend in clinical cancer care. The challenge is to develop technologies aimed at overcoming barriers of paper and pencil format (i.e. difficulties in calculating scores, in showing time trends, in interpreting single patient scores with respect to normative data) meanwhile truly integrated with existing IT tools used in everyday clinical practice, the EMR first among others. Additionally, only electronic assessment of PRMs and their effective integration within the EMR will allow patients’ perspectives to be fully included in the “big data roadmap” [43], a revolution in health care which is about to happen without the contribution of the most important stakeholder: the patient.

Improvement in communication is an expected final result of the PATIENT VOICES project. In addition to the possibility for the patients to express subjective evaluation regarding their condition and experience of care, the use of common platforms and/or PRMs agreed upon by different care providers may also favour communication among clinicians and promote multidisciplinary approach to patient care. However, PRM scores are difficult to communicate in themselves, reflecting the fact that they were developed to report “group level” information in reaserch, rather than individual data in clinical practice. Whereas scores from symptom inventories (i.e. ESAS) are fairly self evident and their communication maybe easy (i.e “Dear Mr. Brown your pain intensity has changed from 7 to 4 (0-10 NRS) after 1 week analgesic treatment”), the communication of composite scale scores, like for example psychological status questionnaires or multidimensional quality of life scales, is more complex and guidelines are missing. For this reason a consistent part of this feasibility project, in particular software development, is dedicated to the study of how PRM scores are reported to stakeholders (clinicians and patients primarily).

For this feasibility study we chose the adoption of a mixed methods research design which will allow to expand and strengthen our study’s conclusions through enrichment of the analysis and findings and the reciprocal validation of data from the two different research frameworks (Creswell & Plano Clark 2007).

This is a single centre project carried out in a comprehensive cancer centre and this may impact on results generalizability. However reports of real-world PRO implementation are limited [44] and to the best of our knowledge this is the first project specifically designed to address implementation barriers and strategies for the systematic assessment of PRMs in cancer care in Italy.

The PATIENT VOICES is an innovative project and as such it may fail to achieve expected impact on the side of technology (i.e. do not reach a full integration of the ePRMs with EMR) or on the side of clinical practice application (i.e. do not reach expected levels of engagement engagement by clinicians and compliance by patients, mainly elderly people or those less educated/less familiar with electronic devices). Nevertheless, the documentation of implementation barriers achieved within the feasibility phase and the development of implementation strategies by the MDST will serve as a starting point for future and more focused interventions aimed at achieving effective ePRMs routine assessment in cancer care and constitute a potential paradigm shift in the management of clinical patient relationships.

Box 1 The PATIENT VOICES project has been developed at the Fondazione IRCCS Istituto Nazional dei Tumori- Milano, a comprehensive cancer centre pioneer in the reaserch field of quality of life in oncology. This was possible also thanks to the scientific contribution of Dott. Marcello Tamburini, former director of the Clinical Psychology Unit as well as founder and first Editor in chief of Health and Quality of Life Outcomes journal.

Marcello strongly believed in the value of patient reported outcome and experience measures for clinical practice in cancer care, and had fully understood the potential of information and communication technology far a long ago. In 2001 he wrote “*A standardised method of evaluating quality of life can help us to understand patient problems to the same degree as standard biological assessments do. This could provide an easy way to anticipate the main problems of the patient. Its function could be similar to that of a thermometer, which detects fever without revealing its cause, the identification of which is the physician’s task. The development of questionnaires in electronic format could help support the clinical use of HRQOL questionnaires, in particular through the use of HTML or similar format with an automatic scoring, a data-entry database and a graphic presentation of the scores. Quality-of-life data could be also used to improve the communication between doctor and patient in order to elicit the patient’s preferences concerning anticancer and symptom therapies.”* [45]

This project is dedicated to his memory (1948–2007).

## Data Availability

Not applicable.

## Abbreviations

DCC: Data Collection Coordinator
EMR: Electronic Medical Record
ePRMs: Electronic Patient Reported Measures
HCPs: Health Care Providers
MDST: Multi-Disciplinary Stakeholder Team
PREMs: Patient Reported Experience Measures
PRMs: Patient Reported Measures
PROMs: Patient Reported Outcome Measures
